# Children’s perspectives of their everyday food practices: insights to inform policy and interventions

**DOI:** 10.1371/journal.pone.0341234

**Published:** 2026-01-27

**Authors:** Sophie Wright-Pedersen, Helen Vidgen, Danielle Gallegos

**Affiliations:** 1 Centre for Childhood Nutrition Research, Faculty of Health, Queensland University of Technology, South Brisbane, Queensland, Australia; 2 School of Exercise and Nutrition Sciences, Faculty of Health, Queensland University of Technology, Kelvin Grove, Queensland, Australia; University of Sydney, AUSTRALIA

## Abstract

Children’s everyday food practices have a profound impact on their physical, mental, cultural and social health and wellbeing. Grounded in social practice theory, the focus of this paper is the examination of the performance of food practices, rather than the practitioner. This approach supports health promoting efforts to move away from victim blaming and instead explore the intersections between individual, social and structural determinants of food practices. With foundations in the New Sociology of Childhood, this article explores eight- to twelve-year-old children’s perspectives of the enablers and constraints to their performances of everyday food practices through a social practice theory lens. Children participated in sequential creative draw-and-tell interviews and Photovoice methods. Through abductive analysis of qualitative data, diverse interlinking configurations of the meanings, materials and competences were attributed by children as either facilitating or constraining food practice performances further impacted by transitioning times, places, social settings and contexts. A case study of food shopping practices was able to present a holistic narrative of how individual, social and structural determinants intertwined across the temporal and spatial dimensions. This study showcases how a social practice led approach that privileges children’s voices can be used to inform more holistic, equitable, engaging and effective health policy and practice that endeavour to impact children’s routine and habitual food practices.

## Introduction

Public health and health promotion fields have increasingly focused on children’s food and their accompanying behaviours as a significant point of intervention to optimise their development and wellbeing, with particular exploration of the enablers and barriers to healthy consumption [1,2]. Much of the research examining children and their food has been garnered from adult viewpoints, but rarely from children, especially those in younger age groups [3,4]. The New Sociology of Childhood, positions children as being competent, capable and active constituents that shape their everyday worlds, thus reorienting the view of children away from objects to be researched and worked upon, towards subjects as part of the research and its’ practical application [5]. Coincidingly, it recognises that adults may not be able to always understand how children experience their worlds (including that of food) and therefore will likely interpret the needs of children differently [5].

Within the adult-dominated literature concerning food, there is a substantive body that frames food and its’ consumption through a medicalised individualistic lens emphasising ‘risks’ associated with food and the body [6,7]. When food is solely rationalised for physical bodily function, aesthetic meanings or sensual pleasure associated with food tends to be ignored as well as any socially constructed inequities of ‘healthy’ food access and culturally diverse meanings [8,9]. As food consumers under this medicalised lens, children are constructed as ‘objects of nutrition’ and the way in which they engage with food problematised and pathologised [10], such as not meeting set dietary guidelines or for generating excess body weight (see for example, [11]). This framing increases the likelihood of children and their families being blamed for undesirable physical health outcomes which may then be reinforced intrinsically resulting in shame [12].

Research focused on macro-environmental determinants of children’s food and their accompanying behaviours has been suggested as a way to address food access and health inequities, steering focus away from individual responsibility [13]. The modification of school food environments towards those more supportive of healthy eating is an example. These macro-environmental approaches, however, have been critiqued as maintaining an individualistic focus through techniques to limit food choices or change individuals’ behaviour whilst ignoring social influences [6]. Thus, when more ‘supportive’ food environments are created, individuals may continue to be blamed for their ‘unhealthy’ actions [12]. More holistic models exist, such as ecological systems theory [14] which presents determinants of children’s food behaviours across individual, interpersonal, community and societal levels. However, the interactions within or between these levels are less explored within research thus limiting insights into how determinants intersect and affect one another (see for example, [15]).

These identified limitations have led researchers to consider how investigations into children’s interaction with food can be framed in ways that consider social and structural inequities whilst not discounting individual agency. Sociological practice theories shift examination to the materials (e.g., funds to acquire food), meanings (e.g., commensality or taste), and competences (e.g., knowledge of situation-appropriate foods) that constitute everyday practices rather than focusing on individual behaviours. Consequently, these theories allow the interconnected and complex nature of food to be illustrated by considering practices as socially constructed configurations of individual, social and structural factors, where individuals, in this case children, are instead viewed as ‘practitioners’ recruited to perform practices [6,16]. By placing practices at the centre of enquiry and privileging the doings and sayings of practices from children’s perspectives, with the underpinning acknowledgement that these are different from adults, this approach shows how social practice theory and child sociology can be complementary. Throughout this study, ‘food practices’ encompass food planning (e.g., creating shopping lists), acquisition (e.g., shopping), preparation (e.g., cooking), consumption (e.g., eating or more specifically as snacking or ‘eating out’) and tidy-up (e.g., managing leftovers) practices. Understanding food practices from the child lens should inform the design of public health food and nutrition policies and programs that align with children’s lived realities. Although there are some applications of practice theory in child-focused literature, such as in the areas of physical activity and lived experiences of obesity (for example, [17,18]), there are limited examples of literature applying a practice-theoretical lens to children’s perspectives of their food practices. A practice-oriented, child-centred approach may reveal why some child-focused public health nutrition efforts fail to gain traction and where modifications to current approaches could be made.

Encompassing a sociology of childhood and social practice theoretical approach, the current research therefore aimed to explore the configurations of Australian children’s everyday food practices from the perspective of eight- to twelve-year-olds in an attempt to better understand what may enable or constrain their performances of food practices across interconnected individual, social and structural domains.

### Theoretical framing

**Fig 1:**
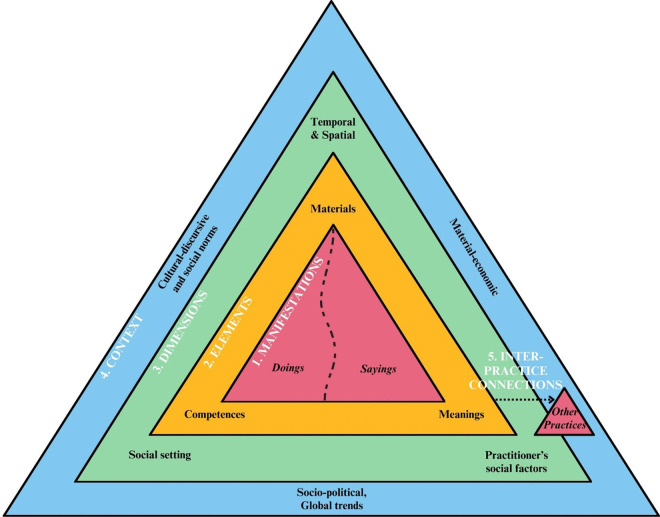
Social practice theory framework adapted from Castelo et al. [21] and Torkkeli et al. [23].

Practices are manifested through actions of ‘doings’ and ‘sayings’ [19]. Practices as described by Schatzki [20](p72) are: *“…Bodily doings and sayings are actions that people directly perform…things people do with their bodies…Sayings, moreover, are a subset of doings, in particular, doings that say something (usually about something)”*. Researchers have proposed theoretical ways of exploring everyday practices, such as Castelo et al. [21] who constructed a framework for ‘zooming in’ and ‘zooming out’ on food practices, inspired by the work of Nicolini [22]. A social practice theory framework is presented in Fig 1 (adapted from Castelo et al. [21] and Torkkeli et al. [23]) showing how food practices may be presented as the configurations of *elements*, dynamically influenced by changing *dimensions* and wider *contexts* as well as forming inter-practice *connections*.

Zooming in on food practices examines the three core interconnected *elements* as proposed by Shove et al. [24], meanings, materials and competences. *Meanings* refer to the social and symbolic significance for why the practice is performed (e.g., ethics, sustainability or health), *materials* concern instruments necessary to perform the practice including the body itself, and *competences* are forms of understanding and practical knowledge necessary to perform the practice.

As these elements recurrently overlap and connect they configure as practices [24]. As per Shove et al. [24](pp14–15), *“practices emerge, persist, shift and disappear when the connections between these elements are made, sustained or broken.”*. The establishment and sustainment of practices is dependent on having constant practitioners of practice (i.e., people) that can perform practices by integrating elements to form practice configurations [24]. Thus, to understand how children are recruited and maintained as practitioners to perform specific food practices, or prevented from becoming or continuing as practitioners, insight is needed into the practice elements (i.e., materials, meanings and competences) and the linkages between them.

The emergence and modification of practices is dynamic, changing over time and space [24]. Therefore, the second component of zooming in on practices explores spatial, temporal, social and practitioner *dimensions* of food practices [21]. *Spatial* relates to the practice setting (e.g., home or dining out), and *temporal* to the time practiced within (e.g., week versus weekend, or everyday routine versus celebratory events). The *social setting* refers to who the practice is performed with (e.g., alone or with family or friends), and the *social factors of the practitioner* relate to the individual performing the practice, for example, age or cultural background. Zooming out on practices examines the connections between practices dependent on the wider *context* in which they are performed [21].

## Methods

### Study setting and design

The data presented in this paper were part of a broader qualitative participatory ethnographic study into children’s perspectives of their food practices. Children’s descriptions of the types of food practices (i.e., food planning, acquisition, preparation, consumption and tidy-up) they were involved in performing across their everyday lives have been reported in detail previously [25]. The current paper reports on the intertwining elements, dimensions and contexts that enable and constrain children’s performances of these food practices.

This study was based in Brisbane, the capital city of the Australian state of Queensland and targeted children of upper-primary school age. Within Australia, most children attend primary school between the ages of four to twelve years old. Children spend approximately six hours a day at school, with two or three structured meal breaks occurring throughout the day. Most Australian schools do not provide school meals, instead children either bring food packed in containers (known as lunchboxes) from home or purchased at a school canteen (or tuckshop). Throughout this paper, brands unique to the Australian context quoted by children have been replaced with generic terms. This study was conducted in accordance with ethical guidelines and was approved by the Queensland University of Technology Human Research Ethics Committee (QUT/UHREC/5274).

### Recruitment

A convenience sampling approach was taken where families with children aged eight to twelve years old residing within the greater Brisbane area were recruited via paid social media advertising (Meta™ platform). Sampling aimed to achieve maximum diversity across age, socioeconomic status and sex. Socioeconomic status was broadly determined using the Index of Relative Socio-economic Disadvantage (IRSD) based on residential postcode, where a low score (i.e., quintile 1) is considered relatively more disadvantaged than a high score (i.e., quintile 5) [26]. Age was based upon date of birth and sex was self-reported by children. For more detailed recruitment methods, see [25].

For each study component, caregivers signed and returned consent forms via email or at interviews after reading study information sheets. Children’s informed consent was obtained for each study component by showing them a short, animated study information video. Children were then asked consent confirming questions (for example, ‘Why are you being asked to participate?’) and given an opportunity to ask questions before being asked to provide written consent. In line with ethical research recommendations, children were given opportunities to assent, dissent, or withdraw at the commencement of each research stage as well as during the research itself [27].

### Data collection

Data were collected from children between September 2022 and January 2023. The first author conducted all interviews and training. All interviews were audio-recorded. Children could choose for their caregivers to accompany them during interviews. In all cases, caregivers were present either at the interview or in the background. Forty-two children (28 families; average age 9.4 years, 52% female, average IRSD 3.57) participated in creative interviews where children were guided through drawing pictures and answering questions according to a semi-structured interview guide. Interviews were conducted at children’s homes (n = 19 families), local public facilities (i.e., libraries (n = 3), university campuses (n = 3)), or using online video conferencing software (n = 3, https://zoom.us/). For more details on data collection methods, see [25].

Twenty-one children (16 families average age 9.6 years, 55% female, average IRSD 4) proceeded to Photovoice. Photovoice was selected as a method both to reduce the power differential between researcher and participant thus empowering children in data collection and also to capture not just the ‘sayings’ of practices but also the ‘doings’. Children were trained in this method and instructed to take photos of food-related activities that they perceived as important to them over a two-week period. Training was conducted in-person (4 children, 3 families) or online (17 children, 13 families, https://zoom.us/). One child/family withdrew for unknown reasons. After two weeks, children and their caregivers returned photos (after reviewing the sensitivity of content) and participated in Photovoice interviews. Interviews were conducted in homes (n = 17 children, 12 families), local libraries (n = 2 children/families) or online (n = 1 child/family, https://zoom.us/). At interviews, children chose photos they wished to talk about and were asked questions by researchers using a semi-structured interview guide. Children and their caregivers chose how their photos could be used in publications (i.e., not at all, with/without blurred faces). Image release forms were signed for photos with identifiable faces.

Creative interviews lasted approximately one hour, and Photovoice interviews lasted approximately 30 minutes. For all interviews, data saturation was achieved when children’s responses to interviewer questions produced limited new data [28].

### Data analysis

All audio data were transcribed verbatim using Descript automated software (https://www.descript.com/) with thematic analysis conducted in NVivo 14 (released March 2023, https://lumivero.com/products/nvivo/). Demographic data were analysed using simple statistics in Microsoft Excel (https://office.microsoft.com/excel).

The research team undertook thematically analysis on qualitative data using an abductive approach [29] to develop a codebook based upon the social practice theory framework (Fig 1). For more detailed codebook development methods, see [25]. Initial coding identified the types of everyday practices that children described, as well as ‘zooming in’ on the food practice elements (applying Shove’s [24] definition) and dimensions, before ‘zooming out’ on the practice contexts. Codes relating to the food practices that children described were defined as ‘macro-food practice’ themes and ‘micro-food practice’ sub-themes informed by conceptual practice theory frameworks [21] and food literacy constructs [30]. In summary, macro-food practices were themed as food planning, acquisition, preparation, consumption and tidy-up. Micro-food practices sit as sub-themes within macro-food practices such as ‘ordering and purchasing food’ under ‘food acquisition’. Codes relating to food practice elements were analysed further as either enabling or constraining to the practice performance. Codes determined through ‘zooming in’ and ‘zooming out’ were charted against micro-food practices through running matrix coding queries in NVivo and presented as a framework matrix [31] (see S1 Table) similar to that presented by Spotswood et al. [32] in examining children’s physical activity practices. This framework matrix then allowed for codes to be presented against the social practice theory framework (Fig 1) to represent how children’s descriptions of their micro-food practices configured and the multiple practice dimensions and contexts these configurations were situated within. Finally, codes were examined across practices to explore how elements transported through food practices and how practices moved between dimensions.

To ensure anonymity, children were assigned pseudonyms by the authors. All primary caregivers were identified as ‘parents’ by children and therefore have been described as such within results.

### Positionality statement

All authors are white, female, Australian-born adults educated within the dietetic profession. The first author was a PhD candidate during the study period and the second and third authors are academic supervisors with backgrounds in qualitative research methodologies. The first author has no children, and the second and third authors are mothers.

## Results

### Zooming in and out on children’s food practices

The framework matrix (S1 Table) summarises how the results of ‘zooming in’ on practice elements (enabling and constraining) and dimensions and ‘zooming out’ on contexts were charted against micro-food practices. The micro-food practices selected for inclusion in the framework matrix were those described by children to a greater extent. Items presented in the matrix are listed in order of the frequency described by children in an attempt to showcase how configurations of elements changed depending on the practice and how practice dimensions and contexts affected practice performances.

### Zooming in: materials, meanings and competences

Children described practice elements (layer two, Fig 1) as both enabling and constraining to food practice performances. Enabling materials commonly described by children across food practices included food and the body. Food as a *material* was present throughout food acquisition, preparation and consumption practices, however, was not necessary for food planning or tidying up. Children described variations in access to and availability of specific types and amounts of food depending on the wider material-economic context, for example food supply, geographic location, or family income, further impacted by the natural environmental context such as flooding events as described by brothers Nakia and Darius.

Interviewer: Is there anything that makes it hard for you to get, eat or make food?

Nakia (boy, age 9): It depends on like if that shop doesn’t have the materials we need. Like the herbs maybe, maybe the stuff that makes it taste better. Like the little details.

Darius (boy, age 11): Sometimes they run out of herbs, especially in the floods, there was less like really less food, especially our favourite bread. …So we had to get like another [bread] …Sometimes I have to get more expensive bread and it was even worse. …and sometimes we have to like, we make sure that our house that we live in … we try and get like a house that has a train station and [a large supermarket chain] near it. …Because we also have a train station near us, like for transport …like easy transport, easy food.

The body as a *material* was present within all food practices and varied depending on those involved in performing the food practice. This included that of the child as well as other practitioners performing the practice. Parents served as a conduit to access other materials, for example, money or food, but were sometimes seen to constrain children’s performance of food practices when they performed practices without children.

Other enabling materials were more specific to the food practice being described. For example, monetary materials (i.e., money, vouchers, other food to trade) were necessary across acquisition practices but not for other food practices; recipes were only used for meal planning and food preparation practices; and specific preparation, consumption and cleaning infrastructure were necessary for respective food practices. As Owen describes, when children did not have access to necessary materials they were unable to perform food practices.

Interviewer: Do you think there’s anything that makes it hard for other kids to eat or get food?

Owen (boy, age 10): Money wise getting food. … if they don’t have a whole lot of money they can’t go to fancy stuff. Us [our family], we just like to save a lot of money, like just Mum and Dad. So that’s why we don’t really go out [to eat] that much. And if we do, sometimes we just cheat and use all the free [fast food establishment] vouchers.

*Meanings* were more specific to individual food practices, and the dimensions and wider context in which these were being performed. For example, in the quote below, Hannah describes how altruistic meanings of caring for animals and the subsequent emotional responses resulted in her actively reducing her meat consumption, whereas learning about the nutritional value and health implications of foods held little meaning and therefore impact on increasing her vegetable consumption.

Interviewer: Why do you think that [learning about sustainable food] changed the way that you were eating and [learning about healthy food] didn’t?

Hannah (girl, age 9): Probably since like the pescetarian thing reached out to me emotionally. Like I feel really bad when I think about animals getting killed for meat. The Australian Guideline to Healthy Eating didn’t really touch me like that did like it didn’t mean that much to me. It was something I was taught, remembered, put in a test, learn something new in Health [class].

… Interviewer: Is there anything that you think would make you care more about the Australian Guide to Healthy Eating?

Hannah: Unless I heard that kids were getting murdered if they didn’t eat their vegetables. I can’t really think of anything that would make me really think about it. It doesn’t mean that much to me.

Similarly, children conveyed a multitude of enabling *competences* that varied across food practices. Children described their knowledge of normative orientations of food practices dependent on the context that they were being performed in, as well as a knowledge surrounding nutritional and monetary value, and food rules and how to navigate them among many others. Skills that children described implementing to perform food practices were plentiful and included food label reading, food ordering, following recipes, negotiating with others, intuitive eating, and using specific infrastructure. Conversely, some children described how lower self-efficacy in food preparation skills and using specific cutlery constrained food practice performances, although this did not always discourage children from attempting the practice.

Interviewer: Do you make the eggs yourself?

Mia (girl, age 12) You can make them in the microwave. It’s pretty easy. …You whisk up the eggs with the whites and yolks in there, and then you put it in the microwave for like a minute and then you stir it around and then it fluffs. It goes fluffy and goes up.

…Interviewer: Are there other foods that you make?

Mia: Well, I do make my [school] lunch so I chop up like fruit and veggies and then put that into containers and put it into my lunch box.

### Zooming in: spatial, social and temporal dimensions

The dimensions in which children performed food practices (layer three, Fig 1) influenced the configurations of practice elements as materials became more or less available, meanings transformed and competence became specific to the practice setting. Compared to the elements of food practices children described dimensions as less varied.

The home environment dominated the *spatial* setting across most food practices including the preordering of school tuckshop and packing of school lunchboxes. Food consumption and to a certain extent acquisition, were the dominating food practices performed by children in schools, however these were more frequently described outside of the school environment.

Children reported their food practices being highly *social*, and consequently, highly enjoyable. Immediate family dominated the social setting as many food practices were performed in the home or children tended to accompany families within other settings (for example, food stores). As the spatial dimension changed so too did the social setting, for example, within schools children were predominantly surrounded, and consequently influenced, by other children when consuming or purchasing foods.

Kane (boy, age 8): I saw my friends eating [kangaroo shaped biscuits] and so I wanted to have them, and I always asked my Mum “Mum, can we get these?”

Although, friends did not appear to have as big an influence on children’s food practices as family members in other settings.

Across the *temporal* dimension children described the majority of their food practices as highly routinised everyday occurrences elicited though phrases including *‘always’* and *‘usually’.* The school day was used as a descriptor of time where weekends and school holidays were described as less structured compared to school days. Non-school days were described as providing more time to perform food shopping and preparing more complex and time-consuming meals. Some food practices, however, specifically occurred around the school day such as packing lunchboxes, ordering tuckshop food, and sharing and trading foods. ‘Special occasions’ were less frequent, non-routine events such as parties, special school tuckshop days, and cultural celebrations. Children described how these events would sometimes facilitate their performance of specific food practices such as meal planning, ordering tuckshop foods, and preparing and consuming specific foods relevant to the special occasion as Lakshmi describes.

**Fig 2:**
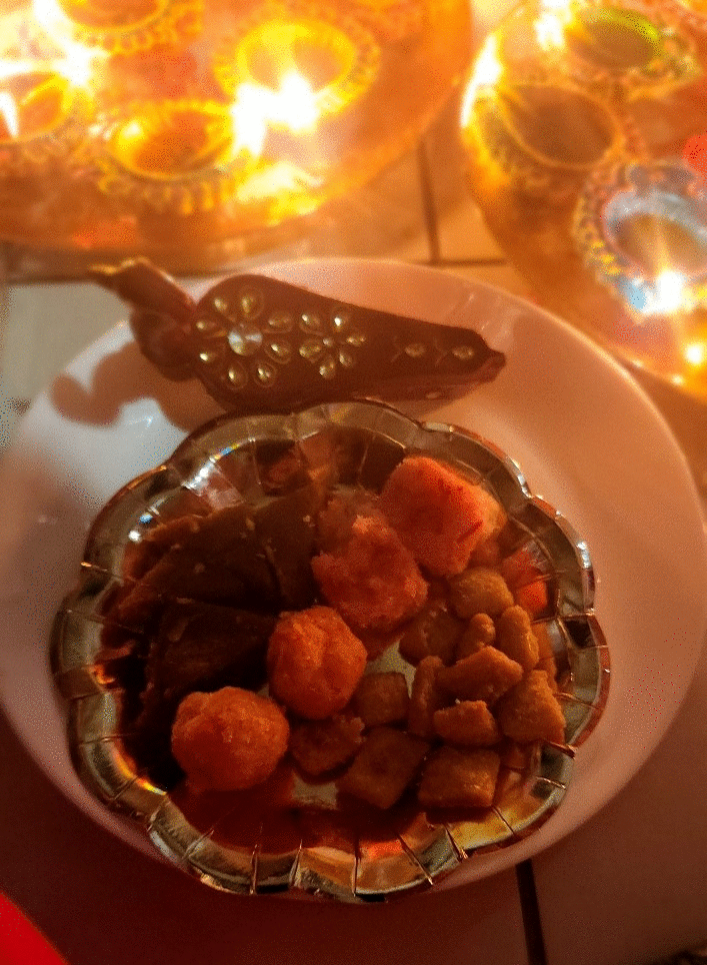
Photo taken by Lakshmi (girl, age 11) of plate of sweets during Diwali celebrations.

Interviewer: And these sweets [referring to photo, Fig 2], do you only have them at Diwali?

Lakshmi (girl, age 11): Well like sometimes maybe, like barely usually. But it’s actually mostly, um, all our festivals of any kind.

Interviewer: Are there other festivals that you would have these?

Lakshmi: Um, yeah, we’ve got at the start of the year around March, we have Holi the colour festival. We’ve got Navaratri…and that during that time my grandma and my Mum do fasting sometimes. And we make foods like sweets like this.

### Zooming Out: Contexts surrounding food practices

Finally, children provided insights into the wider contexts of the food practices they performed (layer four, Fig 1), particularly food acquisition, planning and consumption, and how these contexts interacted. Within the *socio-political context*, children described rules surrounding food instigated by both parents and schools, as well as the impact of COVID-19 bans on the number of foods allowed to be purchased within stores, and international wars on food supply and pricing. Children linked this to the *material-economic context* detailing their understand of the food supply (for example, within stores and tuckshops) as well as the affordability of food, concerning both family income and food pricing, and how this impacted food practices.

Interviewer: Is there anything else on the news that you’ve seen about food?

Luke (boy, age 9): Mm, the cost! I think it was it’s from the war [in Ukraine]. Um, I think [the price] shouldn’t be up because then people have to buy food and it costs so much money and then they don’t have so much money …and then the next time they buy food, they would’ve run out of money.

*Cultural discursive and social normative contexts* described by children revolved around foods appropriate for cultural and celebratory events (for example Christmas, Diwali or birthdays), as well as specific social norms such as vegetarianism. Children also described the social acceptability of specific practices, influenced by those surrounding them, such as foods for school lunchboxes and picking up rubbish within schools. Finally, children described how the *natural environment* impacted food practices such as the seasonality of foods for food gardening and availability when food shopping. Children were also largely concerned on how climatic events such as flooding impacted the food supply and pricing, as well as how their food practices impacted back on the environment such as food packaging, food waste and consuming excessive meat.

Interviewer: Is there anything important about food that you’d like to tell me?

Hannah (girl, age 9): Um, probably like packaging. Like people get a bag of eight packs of mini chocolate chip cookies so they can just stuff a packet in the lunchbox. It’s so much better for the environment and probably cheaper to just get a big bag of cookies and just put a few into lunchboxes …so that there’s less plastic. …Plastic never breaks down. When the chemicals reach into the ground it’s bad for the ground. Nothing good comes of plastic when it’s been used. …Most things come in plastic. Like the chips come in like foil on one side, plastic on the other. It’s really hard to have a lunchbox without using any plastic.

### Practice configuration: A case study of food shopping

**Fig 3:**
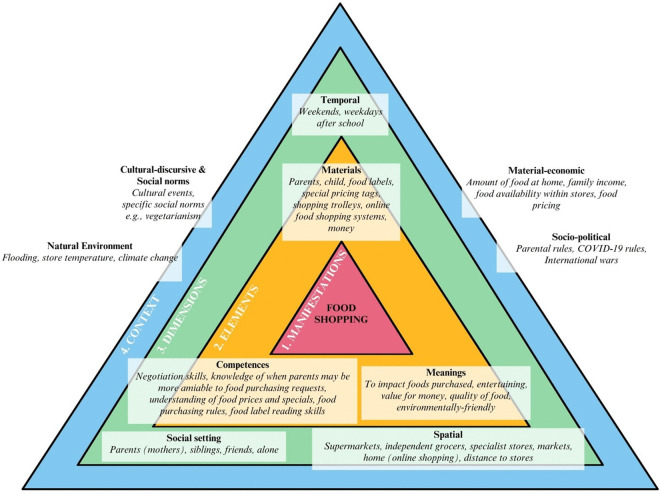
Children’s description of how elements of food shopping practices configured to enable practice performance, and the various dimensions and contexts these were performed in.

Configurations of food practices are formed as elements overlap and become interconnected. The configuration of children’s food shopping practices (as a micro-practice of food acquisition) showcasing the multiple interacting elements, dimensions and contexts is outlined below as an example of this analysis. To do so, information was transformed from the framework matrix (S1 Table) to be presented in Fig 3 as an empirical practice configuration of Fig 1.

Exploring these linkages between materials, competences and meanings may provide insight into how and why children’s food practices emerge, transform, and are maintained across the various dimensions and contexts that the practice is performed within [24].

For example, although parents performed the majority of food shopping practices for households and ultimately made the majority of food decisions *(social)*, children enjoyed going to the supermarket to impact the foods purchased *(meaning)*. This was particularly the case for school lunchbox foods where children tended to have a greater influence.

Interviewer: And do you go with [your parents] if they go to [the supermarket]?

Olivia (girl, age 11): I usually go with Mum if we go to [the supermarket] …I like to choose what I have in my lunchbox. Cause I feel like if my options are there, I can just see what my options are rather than have to think of them in my head.

Children described negotiation tactics they used with parents to influence purchasing and described specific circumstances where parents may be more likely to agree to requests *(competence, social).* Therefore, when children selected foods within stores they used food labels *(material)* to determine the healthiness of the product (for example, via the Health Star front-of-pack Rating system, nutrition messaging and ingredients list), and food pricing to assess value-for-money *(meaning, competence).*

Zara (girl, age 8): I check the Health Star Ratings [front of pack labelling] because if I don’t check that and my Mum checks it herself and it’s like two stars [out of five] she’ll make me put it back and I never get that thing again.

Interviewer: So what, what number of stars can you have?

Zara: Uh, three and a half to five.

Children frequently described a high level of price awareness and detailed knowledge, including an understanding of unit pricing *(competence)*. This was especially the case for food specials where children looked for yellow pricing tags that signalled these *(material)*.

Mia: If there’s something yellow and it says ‘special’, I’d probably ask for it if I wanted it because then it’s more likely [Mum] said ‘yes’.

Interviewer: Does she always say ‘yes’ if you ask for it?

Mia: Most of the time, if it’s on special most of the time. But if it’s not, then maybe, maybe not.

Cost in its own right was not the only contributing factor to children’s assessment of value. Higher value foods were attributed to larger portion or unit sizes *(material, competence)*; if human effort had been put into producing the food *(meaning, material)*; and overall quality or tastiness of the product *(meaning, material)*. Children also considered the seasonality of fruit and vegetables for both increased quality and lower prices *(meaning, competence, natural environmental & material-economic contexts).* Children were also aware of family incomes and how these affected foods bought *(material-economic context)* where they described less expensive foods, for example home brand items, being purchased, and less expensive stores being attended *(competence, spatial)*.

Children were aware that each parent differed in their rules, typical responses or tolerance levels for food requests and were able to use this to their advantage *(competence, social, socio-political context).* However having learnt from experience, many children either did not request food purchases when they knew the answer would be ‘no’ or stopped asking once they were told ‘no’ *(competence)*. Occasionally, children described persisting food purchase requests particularly for lollies and packaged foods, however this was typically over multiple shopping trips rather than in the same one *(temporal)*.

Interviewer: Are there any foods that you want that you don’t ask for?

Ben (boy, age 12): Um, no, not really. We see it and we’re like “Mum, can we please have that?” [Mum says] like ‘yes’ or ‘no’, depending on how much it costs.

Stella (girl, age 9): Like sweets that Mum and Dad don’t usually buy cause we know the answer will be ‘no’.

Interviewer: And if the answer is ‘no’ what do you do?

Ben: Nothing.

Chloe (girl, age 9): Just move on.

Ben: Either beg or just move on. Like “please Mum”

Interviewer: Do you beg for long?

Ben: No. Like, just “please Mum” and if she says ‘no’ then done.

## Discussion

This study demonstrated that children’s perspectives are nuanced and that when supported to do so, they can interpret, understand and communicate the complexities of the food system that impact their everyday food practices. The insights gained from zooming in and out on children’s food practice perspectives include children’s shared and diverse food meanings, the social interaction of practices, and the times and spaces that children engage in food practices. Thus, this child-centred study, grounded in social practice theory, demonstrates that the way in which children perform food-related activities are not only shaped by individual traits such as knowledge or motivation but also by the socio-material arrangements in which food practices occur. Policies and programs that focus narrowly on individual behaviour change risk overlooking the social and structural conditions and the nuanced contextual meanings that shape children’s food practices. Findings may therefore inform how food interventions and policies aimed at transitioning children’s food practices in specific ways may be able to be better adapted to address more meaningful and influential factors [24,33]. Food and nutrition policies that focus on weakening and eventually breaking links between practice elements (i.e., materials, meanings and competencies) may lead to new pathways being formed for new connections and novel practices to emerge and take over, such as more nutritious food consumption [24]. Reframing health promotion messaging directed at children and families, as an example, to integrate and emphasise meanings important to children’s performances of food consumption practices may complement existing policy and intervention work. Findings also point to the settings in which to direct and deliver policy and interventions as the home environment encompassing immediate family member involvement was predominantly where food practices were performed and influenced compared to schools where many children’s food policy and interventions are delivered [34,35].

While the application of a practice-theoretical lens can provide a framework to understand the intersecting individual, social and structural factors impacting children’s recruitment to food practices, privileging their voices challenges traditional food perspectives. The plethora of meanings children attributed to food practice performance, such as emphasis on taste and commensality, counters the problematisation of children’s food unhealthy practices asserted via medicalised ‘risk’ narratives, for example unhealthy food consumption [7]. Within this study, children’s association with the medicalised language of food was in the presence of parents and other adults such as reading food label nutrition information before requesting purchases. Children navigated healthy foods within other food meanings, describing healthy food as fitting within their diets via a position of ‘balance’ almost equally with that of unhealthy foods. This language of ‘balance’ as an equivocal concept has also been recorded within the context of Australian adolescents’ food descriptions [36], highlighting young people’s diverse food messaging interpretations compared to that of professional institutions (see for example, [37]). Further, children rarely described competence as a barrier to performing their everyday food practices. This is in contrast with knowledge and skill as the subject of much nutrition action directed at children by adults (for example, [38]), which tends to frame children as ‘adults in the making’ rather than their current selves, subsequently comparing children’s competency to that of adults. Instead, the integration of children’s perspectives within food and nutrition policy and program material may elevate a strength-based competence framing where children are assessed on what they can do rather than what they cannot.

In moving away from negative, blaming and stigmatising discourses, the integration of children’s perspectives provided a positive reframing of the social nature of their food practices. Although previous adult-centric food shopping studies have negatively positioned children as in conflict with adults, ‘pestering’ or using coercive power [39,40], children in this study described how they negotiated purchases with parents and would cease requests when told ‘no’. This is supported by other studies that have observed parent-child food purchasing interactions within stores as pleasant experiences [41,42]. Observational studies however have limited ability to provide the level of insight described by children in this study with findings supported by previous literature that has sought children’s first-hand food experiences [43]. Children’s perspectives, through a practice-theory lens, therefore, allow for the reframing of food requests as a means of exerting their agency over foods brought into the home, rather than a negative occurrence of children attempting to be “deviant” [44].

### Strengths and limitations

The presence of adult researchers throughout the entire study and the subsequent unequal power dynamics that likely resulted with children should be considered. Strategies to mitigate these included the use of developmentally appropriate and engaging resources and tasks. This was complemented by locating interviews in children’s spaces such as their homes, or neutral locations such as community libraries that children had previously visited.

Practices and cohorts of practitioners performing practices constantly change over time and place, and as such the results of this study are relevant to the specific cohort that participated and the time in which data were collected. The sample included in this study were likely biased towards being more interested, competent and involved in food practices. Thus research with other cohorts, and within other contexts is encouraged to understand varying influences, where longitudinal studies may give insight into why specific practices become embedded in children’s food worlds while others disappear. Further to this, the integration of this research within existing or new research conducted with caregivers, policy makers and food system actors may present a more comprehensive picture of intersecting everyday food practices and how to sustainably modify these.

## Conclusion

Through a sociological practice-oriented view of food which privileged the voices of upper primary-school aged Australian children, configurations of children’s everyday food practices that enabled or constrained performances were able to be understood. This approach was able to present individual, social and structural determinants intertwining across temporal and spatial dimensions of food practices, bridging the agency-structure divide. Further, in amplifying children’s voices, a plurality of socially and structurally driven meanings attributed to food practice performance were revealed shifting focus away from solely considering food as a means to reduce risk portrayed through physical manifestations of health. Implementing this approach throughout children’s food studies may inform more equitable, engaging and effective interventions that meet their needs, priorities and contexts. Ultimately this may assist in reducing blame and stigma directed at children and families.

## Supporting information

S1 Table Framework matrix summary of children’s descriptions of their everyday food practices. Items are listed in order of frequency as described by children.
(DOCX)

S2 File COREQ checklist. (PDF)
