# Supplementary material for: Children’s perspectives of their everyday food practices: insights to inform policy and interventions
Source: PLoS One. 2026 Jan 27;21(1):e0341234. doi: 10.1371/journal.pone.0341234 (PMC12843533; doi:10.1371/journal.pone.0341234)
Supplement: S1 Table — Items are listed in order of frequency as described by children. (DOCX) [file pone.0341234.s001.docx]

S1 Table: Framework matrix summary of children’s descriptions of their everyday food practices. Items are listed in order of frequency as described by children.

|  | Macro-food practices | Planning | Acquisition | | | | Preparation | | Consumption | | Tidy-up |
| --- | --- | --- | --- | --- | --- | --- | --- | --- | --- | --- | --- |
|  | Micro-food practices | *Meal Planning* | *Ordering and purchasing food*  *(food shopping)* | *Ordering and purchasing food*  *(from school tuckshops)* | *Sharing and trading foods at school* | *Growing food* | *Preparing food to eat within the same setting* | *Packing lunchboxes to transport food from home to school* | *Consuming everyday meals* | *Consuming at special occasions* | *Cleaning up* |
| Elements of practice | **Materials** *(enabling)* | - Parents *(as bodily materials)* - Child *(as a bodily material)* - Recipes (i.e., cookbooks) and home-meal kits - Online search engines and websites for meal inspiration | - Parents - Child - Money (via parents) and online food shopping systems - Foods available in stores - Food nutrition labels and pricing tags - Shopping trolleys and baskets - Safety and infrastructure to attend store alone (e.g., quiet streets) | - Child - Parents - Tuckshop convenors *(as bodily materials)* - Money or tuckshop cards (own savings or via parents) - Foods available in tuckshops - Online ordering systems (websites and mobile applications accessible via parents) - Canteen menus | - Child - Possessing foods willing to trade - Food labels listing allergens | - Child - Parents - Seeds to plant, or established fruit trees - Animals to produce foods (e.g., chickens for eggs) - Water and hoses - Pots and garden beds (i.e., space to plant) - Gardening gloves | - Child - Parents and other adults (e.g., grandparent, carer) - Unprepared foods - Preparation infrastructure e.g., microwaves, knives, toasters, blenders, kettles, ovens, stoves - Recipes (cookbooks, food packages) - Electricity - Time | - Child - Parents - Foods appropriate for school lunchboxes - Preparation and storage infrastructure i.e., food preparation equipment, lunchbox, school bags, other containers, ice-bricks - ‘Snack boxes’ in refrigerators and cupboards - Time | - Child - Ready-to-eat food, including takeaway foods, in specific forms (e.g., cut-up) and quantities - Consumption infrastructure (i.e., knives, forks, plates) - Dining infrastructure (i.e., table, chairs) - Time | - Child - Foods appropriate to the special occasions (e.g., cake for birthdays), sometimes in various formats and colours | - Parents or other adults - Child - Items to clean - Cleaning infrastructures e.g., dishwasher, sink, bins |
|  | **Materials** *(constraining)* | - Parents or other siblings performing meal planning instead | - Parents performing without children (e.g., during school hours) |  | - Not having desirable foods to trade - Foods without labels (e.g., homemade foods) | - Plants that are difficult or take a long time to grow | - Less safe preparation equipment, i.e., heat producing such as ovens - Children not available (e.g., at school) |  | - Whole, hard foods - Children’s small mouths, loose teeth, braces |  |  |
|  | **Meanings** *(enabling)* | - For increased choice and variety in subsequent food practices - Altruistic (sustainability and ethical i.e., reduced meat consumption) or health ideals | - To impact foods purchased (for taste, quality, environmentally friendly options) - Value for money - Feelings of enjoyment | - Novelty of foods in tuckshops compared to usual foods - Tasty foods - Value for money (e.g., lower cost) - Choice and independence - Convenience and efficiency - Backup option (e.g., when lunches were left at home) - Commensality (e.g., to share with other children) - ‘Balance’ of more and less nutritious options | - To acquire preferable foods based on taste - Commensality and enjoyment - To avoid punishment for not eating lunch foods | - Higher quality and tastiness than store bought produce - Feelings of enjoyment and pride - To increase food supply - To learn about how food is produced | - Choice and autonomy in quality, variety and tastiness of foods - For satiation - Feelings of enjoyment - Feelings of responsibility - To build food preparation skills | - Choice and autonomy in quality, variety, amount and tastiness of foods - Convenience - To build food preparation skills - ‘Balance’ of more and less nutritious options | - Taste and texture - Acceptable food quality (e.g., home grown, not bruised) - Variety within and between meals - Higher perceived food value and to gain more valuable foods (e.g., dessert) - Feelings of happiness, nostalgia, enjoyment and entertainment - Feelings of commensality, love and care - Physiological responses (e.g., satiation, energy, regulate body temperature, health implications) - Convenience of foods - Choice of foods - Altruistic (e.g., reduced food waste, animal caring) - Safety & hygiene | - Feelings of fun, excitement, enjoyment and entertainment - Feelings of commensality and nostalgia - Social and cultural significance - Novelty | - Cleanliness for future use - Altruistic (e.g., pick up rubbish for planetary care, sense of responsibility) - Convenience |
|  | **Meanings** *(constraining)* | - Feelings that meal planning (i.e., decision making) is laborious | - Feelings of boredom - Trust in parents to purchase preferred foods - Feeling cold in stores | - Having to wait for tuckshop orders when ordering directly or collecting from school | - Fear of getting in trouble from teachers - Fears of other children having anaphylactic events - Minor concerns around hygiene risk (e.g., COVID-19) - Value the effort parents put into preparing lunchboxes - Enjoyment of current foods or were not hungry | - Feelings of failure in growing foods | - Feeling unsafe in food preparation practices - Feelings of unenjoyment | - Feelings of unenjoyment | - Physiological responses i.e.., over-satiation, sometimes impacted by medication - Fear of not enjoying the taste of foods - Feelings of anger and disgust |  | - Feelings of unenjoyment |
| Elements of practice (cont.) | **Competence**  *(enabling)* | - Knowledge of usual and preferred foods - Knowledge and skills in understanding existing availabilities of food at home (e.g., checking refrigerator contents), and practicalities of meals that can be prepared - Ability to predict potential hunger levels to determine amount of food necessary | - Knowledge of food purchasing rules and how to navigate them - Negotiation skills and knowing when parents may be more amiable to requests (e.g., cheaper foods, more nutritious foods, foods for lunchboxes, closer to special occasions) - Knowing when to not request purchases & when to stop requests - Understanding of value and vigilance of food pricing - Label reading skills (e.g., health star ratings, ingredients) - Knowledge of which supermarkets are cheapest & food availability - Knowledge of foods at home and what needs to be purchased | - Negotiation skills and knowing when parents may be more amiable to requests (e.g., special tuckshop days, birthdays, nutritious options) - Ability to assess food value (i.e., comparing food price against amount of food, cost of similar foods in supermarkets, quality and tastiness of food, amount of human effort put into making the food, & perceived overhead costs) - Nutrition knowledge and awareness of modifications for healthier tuckshop options - Ability to use online ordering systems and top-up payment systems | - Knowledge and understanding of school rules and how to navigate them - Lack of understanding of school rules and why they should be followed - Contextualised food value knowledge & negotiation skills to acquire higher value foods (e.g., ‘two for one’ bargaining techniques) - Knowledge of who to safely trade with, and what foods, to avoid allergic reactions - Label reading skills to check for allergens | - Knowledge and skills to grow foods including watering, planting, and harvesting - Knowledge of environment necessary to grow foods e.g., seasons, shade | - Spectrum of simple to highly complex preparation skills, and ability to adapt techniques - Knowledge of recipes and methodology | - Spectrum of simple to highly complex preparation skills - Ability to predict hunger levels and adapt portion sizes - Ability to pack foods that meet school and parental food rules - Negotiation skills to influence parental packing decisions - Knowledge of nutritious foods - Knowledge of when to pack an ice brick | - Knowledge of what foods are appropriate for specific contexts - Intuitive eating skills including adapting meals (type of food, portion sizes or timing of consumption) to satiate or prevent hunger - Skills in utilising eating utensils - Knowledge of school and parental rules and how to navigate - Understanding of impact of consumption practice on the environment - Healthy food knowledge | - Knowledge of what foods are appropriate for specific context/event | - Cleaning skills, knowledge of how to stack and use dishwashers |
|  | **Competence**  *(constraining)* |  |  |  |  |  | - Low self-efficacy in food preparation skills necessary to perform tasks (e.g., cooking on the stovetop, taking food out of hot ovens) |  | - Low self-efficacy in skills required to use chopsticks; and knives and forks simultaneously |  |  |
| Dimensions of practice | **Spatial** | - Home - In stores | - Supermarkets - Independent grocers - Specialist stores - Markets - Home (online shopping) - Distance to stores | - Home (via online) - School (directly from tuckshop) - In cars (on the way to school via online) | - School (playground) | - Home - Schools | - Home (kitchens) - Other homes (e.g., grandparents, friends) - Afterschool care - Schools - Girl Guides | - Home (kitchens) | - Home (dining table, kitchen benches, living spaces) - School (eating areas) - Afterschool care | - Home - Other homes (e.g., grandparents, friends) - Community settings - Restaurants, fast-food establishments | - Home - School |
|  | **Social** | - With parents | - With parents or whole families - Occasionally with siblings and friends - Occasionally alone - Impact of needs and food preferences of other family members | - With parents (via online) - Alone (via online) - From tuckshop convenors (in-person) - With or for friends (in-person) - Impacted by decision-makers of canteen menus | - With friends - With other children - Occasionally in the presence of teachers | - Alone or with siblings - With other children (at school) - With parents | - With parents - Alone - For others | - Alone - With siblings or parents - For other siblings | - With entire immediate family - With friends and other children - Alone - With other adults (e.g., teachers) | - With immediate and extended family - With friends - With wider community | - With siblings - With family - Alone - In the presence of teachers |
|  | **Temporal** | - When parents were planning shopping trips or performing online food ordering - Routinised & designated times (e.g., once a week) - Special occasions (e.g., birthdays) | - Semi-regularly (e.g., every one to two weeks) - Weekends (larger shops) - Weekdays after school (top-up shops) | - School days - Routinely (e.g., daily, once a week) - Special occasions (e.g., birthdays, end of school term, special tuckshop days) | - School meal breaks - Routinely | - When at home - During school hours | - Meals & snacks - Weekdays & weekends - School holidays - Special occasions - Historical experiences | - Morning or afternoon before next school day | - Everyday routinely - Meals and meal breaks - Historical experiences | - Infrequent - Celebratory events - End of school term/school holidays | - After meals (particularly dinner) |
| Context of practice | **Socio-political** |  | - Parental rules (e.g., allocated number of items to freely choose per shop or per week) - COVID-19 restricting items purchased - International wars (affecting food pricing) | - Parental rules and school systems (e.g., banned foods lists for less nutritious options) | - School rules banning sharing and trading foods - Permission from parents to trade/share | - COVID-19 restricting items purchased | - Parental food rules (e.g., use of less safe appliances) | - School rules (e.g., no nuts) - Parental food rules (e.g., nutritious options) | - Parental rules (e.g., nutritious options) - School rules (e.g., time and spaces allocated for eating) |  |  |
|  | **Material-economic** |  | - Family income - Food pricing - Food availability within stores (supply) - Foods available at home | - Family income - Foods available at home - Food pricing within tuckshops - Food availability within tuckshop - Opening days of tuckshop |  | - Lack of foods available in food stores (COVID-19 impacts) | - Foods available at home | - Foods available at home | - Foods available at home |  |  |
|  | **Cultural-discursive & social norms** |  | - Cultural events - Specific social norms (e.g., vegetarianism) | - Specific social norms (e.g., vegetarianism) |  |  |  | - Social norms of school food environment | - Food rituals - Specific social norms (e.g., vegetarianism) | - Cultural norms - Celebratory norms | - Social norms of picking up rubbish at school |
|  | **Natural Environment** |  | - Climate change events, e.g., flooding - Seasonality of foods - Store temperature | - Crows stealing foods brought from home |  | - Seasonality of foods |  |  | - Outdoor temperature and climatic conditions |  | - Climate change and natural resources |
